# Estimated medical costs of methicillin-resistant *Staphylococcus aureus* infection classified by polymerase chain reaction-based open reading frame typing in Japan

**DOI:** 10.3934/microbiol.2022034

**Published:** 2022-12-19

**Authors:** Tomokazu Shoji, Ryusei Muto, Ryoko Sakai, Hiroki Matsumura, Takashi Uchida, Fumihiko Kitta, Osamu Inoue, Keishi Kawata, Manabu Akazawa

**Affiliations:** 1 Department of Pharmacy, University of Yamanashi Hospital, 1110, Shimokato, Chuo, Yamanashi 409–3898, Japan; 2 Department of Public Health and Epidemiology, Meiji Pharmaceutical University, 2–522–1, Noshio, Kiyose, Tokyo 204–8588, Japan; 3 Division of Infection Control and Prevention, University of Yamanashi Hospital, 1110, Shimokato, Chuo, Yamanashi 409–3898, Japan; 4 Department of Clinical Laboratory, University of Yamanashi Hospital, 1110, Shimokato, Chuo, Yamanashi 409–3898, Japan

**Keywords:** MRSA, administrative claim database, PCR-based open reading frame typing, healthcare cost, inverse probability of treatment weighting

## Abstract

This retrospective, observational cohort study investigated the economic impact of genotype by classifying methicillin-resistant *Staphylococcus aureus* (MRSA) by using the polymerase chain reaction-based open reading frame typing (POT) method. Using administrative claims and bacteriological data for April 2016 to March 2021 from the University of Yamanashi Hospital, we ascertained the POT1 numbers and classified MRSA as either “hospital-derived” or “community-derived”. We defined MRSA-associated medical practices and estimated the associated medical costs. After applying inverse probability of treatment weighting (IPTW)-based adjustment for patient characteristics between the two groups, we estimated the differences in medical costs during the “total therapy period” (defined as the interval from specimen submission to Day 42 after the susceptibility report) and the “definitive therapy period” (defined as the interval from susceptibility reporting to Day 42). Among the 135 MRSA-infected patients, 54 and 81 were classified as having hospital-derived and community-derived MRSA infections, respectively. Significant differences in patient characteristics were observed with regard to age (*p* = 0.0478), sex (*p* = 0.0422), surgery (*p* = 0.0349), chemotherapy (*p* = 0.0457) and immunosuppressive drug use (*p* = 0.0222). The median duration of the definitive therapy was 29 and 27 days, and the mortality rate during this period was 11% and 5% for the hospital-derived and community-derived types, respectively. After IPTW-based adjustment, the medical costs for the total therapy period were 324,480 and 296,462 Japanese yen (JPY) per patient for the hospital-derived and community-derived types, respectively, whereas the medical costs for the definitive therapy period were 279,635 and 256,542 JPY per patient for the hospital-derived and community-derived types, respectively. No statistically significant difference was detected (*p* = 0.5813 and *p* = 0.6355, respectively). In this study, MRSA healthcare costs were compared according to the POT scores, and no statistically significant differences were observed between hospital-derived and community-derived MRSA infections.

## 1. Introduction

Antimicrobial resistance is a significant global threat and increases the healthcare cost burden [1]–[4]. Methicillin-resistant *Staphylococcus aureus* (MRSA) has been detected in the majority of healthcare facilities in Japan [5],[6], and the nosocomial transmission of MRSA has been associated with adverse events and increased medical costs [7]. Therefore, the importance of infection control, including proper hand hygiene practices, has been emphasized [8]. To be clinically classified as a nosocomial infection, MRSA should be detected after 48 hours of hospitalization, whereas MRSA detected before 48 hours is classified as a community-acquired infection [9],[10].

The polymerase chain reaction (PCR)-based open reading frame typing (POT) method, which amplifies and scores the presence of specific MRSA genes, is one of the epidemiological analysis methods used to determine the spread of MRSA [11]. POT is a molecular typing tool that was developed for nosocomial infection control to facilitate rapid identification of the bacterial genotypes when nosocomial infection is suspected and clarify the mode of in-hospital transmission [12]. Pulsed-field gel electrophoresis (PFGE) and multi-locus sequence typing (MLST) were used for molecular epidemiological analysis to confirm whether the genotypes of the isolates were identical. Although these methods are suitable for identifying strains in a limited area or comparing strains between countries, because of their limited reproducibility and several days required to obtain the results, PFGE and MLST are unsuitable for identifying the spread of drug-resistant strains in a clinical setting. Compared to these conventional methods, POT is characterized by a shorter analysis time and relatively lower costs of equipment and reagents [13],[14].

The discriminatory effect of the POT score for MRSA has been reported previously [15],[16]. Staphylococcal cassette chromosome *mec* (SCC*mec*) is used to differentiate between hospital-derived and community-derived strains, as hospital-derived strains have types I, II or III SCC*mec*, whereas community-derived strains have SCC*mec* types IV or V [17]. Maeda et al. reported that the POT-kit successfully predicted the SCC*mec* type in 54 strains (95%); moreover, the results of POT1 score-based typing were highly concordant with those of SCC*mec* typing. Therefore, POT can be used to classify hospital-derived and community-derived MRSA by predicting the SCC*mec* type [18]. Ogihara et al. reported the clinical and molecular characteristics of MRSA strains isolated from outpatients in Japan and defined POT1 = 93 as hospital-derived MRSA and POT1 = 104, 106 or 108 as community-derived MRSA [14]. Table 1 presents the characteristics of each type [19]–[21]. Hospital-derived MRSA is highly resistant to several non-beta-lactam antimicrobial agents (quinolones, aminoglycosides, macrolides, etc.) [22]. In contrast, a high percentage of community-derived MRSA is toxin-producing and called the Panton-Valentine leukocidin (PVL)-producing strains [16]. In recent years, in areas where community-derived MRSA clones are highly prevalent and established, for example, USA300 in the USA, community-derived MRSA strains are beginning to supplement or overtake traditional hospital-derived MRSA strains in hospital-associated infections [23]. Although both types of MRSA have their individual characteristics, treatment selection is not based on the POT score, and the outcome and economics of such treatment remain unclear.

Previous studies have compared POT scores with the results of drug-susceptibility tests [14],[24], but reports of MRSA classification by POT scores and estimation of the medical costs are limited. In this study, we classified MRSA by using the POT score, and the relationship between the POT score and medical costs was examined to clarify the impact of genotype on treatment, as this information can facilitate nosocomial infection control.

**Table 1.** Characteristics of hospital-derived MRSA and community-derived MRSA.

	Hospital-derived MRSA	Community-derived MRSA
Clinical definition	Isolated from hospitalized patients	Isolated from healthy people in the community
Bacteriological definition (SCC*mec* classification)	Type Ⅰ, II, Ⅲ	Type IV, Ⅴ
Major clones	New York/Japan	USA300
Toxins	Various toxins	Characterized by leukocytolytic toxin (Panton-Valentine leukocidin: PVL)
Place of endemicity	Hospital-associated	Community-associated (e.g., schools, homes, etc.)
Age	Mainly elderly	Mainly young
Site of infection	Various organs	Mainly skin and soft tissues
Drug susceptibility	Multidrug resistance	Relatively sensitive
Treatment course	Refractory	Good response

## 2. Materials and Methods

### 2.1. Study design and data source

This single-center, retrospective, observational cohort study was conducted by using administrative claims, discharge summary and bacterial testing data from the University of Yamanashi Hospital. The administrative claims and discharge summary data were based on the Diagnostic Procedure Classification (DPC) data, and the bacterial data were recorded at the hospital's bacteriology laboratory. The University of Yamanashi Hospital is a 618-bed university hospital that has undertaken calculations of the medical fee for infection prevention of control type 1 since 2012 and the medical fee for antimicrobial stewardship teams since 2018.

The DPC data recorded payment information according to diagnostic classification and include basic patient information (e.g., age, sex, disease, the International Classification of Disease 10th revision [ICD-10] code), medical treatment (e.g., prescription, examination, surgery, procedure), codes and cost. The bacteriological test data included the specimen receipt date, specimen report date, specimen material, detected bacterial name and POT score.

The POT method was performed by using the Cica Geneus Staph POT KIT (Kanto Chemical Co., Ltd.) with two sets of multiplex PCR, as according to the manufacturer's instructions, and the presence of 22 open reading frames was determined from the obtained agarose electrophoresis images. The amplified products detected by the POT method were classified as POT1-3, and the POT type was determined by calculating the sum of the POT coefficients based on the presence or absence of bands of the target size [25],[26]. The frame detection results and the combination of genomic islet possession patterns correlate with MRSA clones. Therefore, MRSA clones can be estimated from POT1 values, which enables classification using SCC*mec* [12].

In this study, the POT1 score could predict the type of SCC*mec* based on previous reports [14],[27]. The analysis was performed by defining a case with a POT1 score of 93, which predicted SCC*mec* Ⅱ, as hospital-derived MRSA, and cases with POT1 scores of 104, 106 and 108, which predicted SCC*mec* Ⅳ, as community-derived MRSA (Table 1).

### 2.2. Inclusion and exclusion criteria

In this study, we evaluated nosocomial infections in patients who had been infected with MRSA strains [9],[10]. We enrolled patients aged 18 years or more who were admitted or discharged from the University of Yamanashi Hospital between April 2016 and March 2021, had MRSA detected in any specimen on bacteriological examination conducted on or after the fourth day of admission and underwent a POT test based on a suspicion of nosocomial infection. If MRSA was detected more than once per hospitalization episode, the date of the first MRSA specimen submission was used as the index date for the analysis. We excluded the patients who met the following criteria from the study population: 1) patients for whom some or all of the administrative, claims or bacteriology data necessary for analysis were missing after the reporting of detected MRSA, and 2) patients for whom data could not be collected after the date of reporting of the detected bacteria.

### 2.3. Definition of variables

The variables calculated from the DPC data included the following: age, sex, ambulance use, length of hospital stay before specimen submission, the Barthel Index at admission, the Charlson Comorbidity Index, duration of antimicrobial therapy before specimen submission, death at discharge and medical costs.

The following variables before specimen submission were obtained from the DPC data: antimicrobial administration, surgery, chemotherapy, immunosuppressive drugs, granulocyte colony stimulating factor (G-CSF) preparation, blood transfusion, central venous catheter use, drain use, radiation therapy, dialysis, bronchoscopy, intensive care unit (ICU) admission and reason for hospitalization.

The following variables were obtained from bacterial data: specimen material, specimen submission date, bacteria detected, bacterial detection report date and the POT score.

### 2.4. Identification of MRSA infection cases

For determination of drug susceptibility, a minimum inhibitory concentration value of at least 4 µg/mL for oxacillin or 8 µg/mL for cefoxitin was determined as MRSA based on the Clinical Laboratory Standards Institute M100–22. The detection of MRSA alone does not determine whether a patient is infected or a carrier of the organism. Therefore, based on a previous study, we defined a patient as an MRSA-infected case if they met the criterion of antimicrobial therapy initiation within 10 days from the date of bacteriological examination when MRSA was detected and was receiving antimicrobial therapy for three consecutive days or more [1].

### 2.5. Definitions of treatment periods

The empirical therapy period was defined as the time between the date of specimen submission and the date of bacterial reporting. The definitive therapy period was defined as 42 days, starting from the bacterial reporting day. The total therapy period was defined as the time between the empirical therapy period and the definitive therapy period. The therapy period was based on the recommended treatment period for complicated MRSA infections [28]. Observation was discontinued on the day of patient discharge or death during the observational period.

### 2.6. Definition of MRSA-related medical practices

We defined MRSA-related medical practices as described in our previous study, as follows [29]. First, the number of medical procedures performed before and after the detection of MRSA in patients with MRSA infection was summed, and those that were performed more than twice as many times after the detection of MRSA infection were extracted. Second, medical practices that corresponded to medical management, medication, injection, treatment, examination and diagnostic imaging were included according to the categories indicated by the medical fee scale. Medical practices that were considered as not directly related to the treatment of infectious diseases were excluded, such as basic medical fees, home medical care, rehabilitation, specialized psychiatric therapy, surgery, anesthesia, radiation therapy and pathological diagnosis. The medical practices selected by the above-mentioned method were defined as MRSA-related medical practices. Injectable antimicrobial agents classified as “J01” in the Anatomical Therapeutic Chemical Classification Level 3 classification proposed by the World Health Organization were included in MRSA-related medical practices. Oral and topical antimicrobial agents were excluded from calculation in this study.

### 2.7. Outcome definitions

The duration of hospitalization was calculated as the median of the total and definitive therapy period. Mortality was calculated as the mortality rate during the definitive therapeutic period. The average number of MRSA-related medical procedures (times/patient-day) was calculated as the number of procedures. The antimicrobial use density (AUD) in each group was calculated as the amount used per 100 days during the empirical and definitive therapeutic periods. The costs of MRSA-related medical practices and antimicrobials were calculated as medical costs. The fees are summarized in Japanese yen (JPY, 1 USD = 110 JPY) by using each year's medical fee points.

### 2.8. Statistical analysis

The outcomes included as indicators of the clinical and economic burden of MRSA were the length of stay in hospital, in-hospital mortality and medical costs for the total therapy period and definitive therapy period. The study sample was first divided into hospital-derived MRSA and community-derived MRSA groups. The baseline characteristics and outcomes of the two groups were compared by using chi-square or Fisher's exact tests for categorical data and the student's *t*-test or Welch's *t*-test for continuous data, as appropriate.

To adjust for factors that might affect the comparison of hospital-derived and community-derived MRSA, 19 variables (i.e., age, sex, ambulance use, length of hospital stay before specimen submission, the Barthel Index, the Charlson Comorbidity Index, antimicrobial administration, duration of antimicrobial therapy before specimen submission, surgery, chemotherapy, immunosuppressive drugs, G-CSF preparation, blood transfusion, central venous catheter use, drain use, radiation therapy, dialysis, bronchoscopy and ICU admission) were used as covariates to calculate the propensity score by using a logistic regression model, and inverse probability treatment weighting (IPTW) was used to compare medical costs. Statistical significance was set at *p* < 0.05. All statistical analyses were performed using Statistical Analysis System 9.4 (SAS Institute, Cary, North Carolina, USA).

### 2.9. Ethics approval of research

This study was approved by the Ethical Review Committee of the University of Yamanashi Hospital (No. 2510) and conducted in accordance with the Ethical Guidelines for Epidemiological Research established by the Japanese government, which stipulates the requirements for protecting patient anonymity. Informed consent was obtained in the form of opt-out, as hosted on the hospital website.

## 3. Results

### 3.1. Demographic characteristics

**Figure 1.**
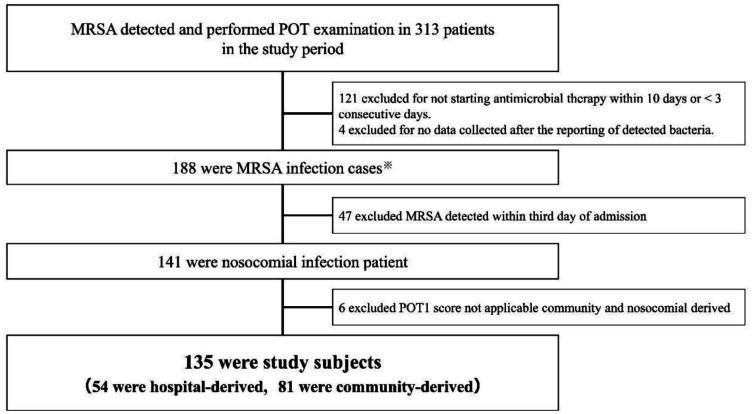
Flowchart depicting participant enrollment. Abbreviations: MRSA, Methicillin-resistant *Staphylococcus aureus*; POT, Polymerase chain reaction-based open reading frame typing. ※Started antimicrobial therapy within 10 days from the date of bacteriological examination when MRSA was detected and had received antimicrobial therapy for 3 consecutive days or more.

As shown in Figure 1, among the 135 participants, 54 and 81 were categorized as hospital-derived and community-derived MRSA types, respectively. Details of the POT numbers for both groups are described in Supplementary Table 1. The most common reasons for hospitalization were neoplasms (44%) for hospital-derived MRSA and cardiovascular disease (30%) for community-derived MRSA, respectively. Table 2A shows the patient characteristics for hospital-derived MRSA and community-derived MRSA, including age (65.8 years vs. 70.7 years; *p* = 0.0478), sex (male: 81.5% vs. 65.4%; *p* = 0.0422), surgery (63.0% vs. 44.4%; *p* = 0.0349), chemotherapy (24.0% vs. 11.1%; *p* = 0.0457) and immunosuppressive drug (46.3% vs. 27.1%; *p* = 0.0222). Respiratory-related samples were the most common specimen material detected in both groups (64.9% vs. 50.7%). The median (25–75%) overall duration of treatment was 32 days (18–44 days) for hospital-derived and 30 days (20–44 days) for community-derived (*p* = 0.9332) MRSA infections. The median (25–75%) duration of definitive therapy was 29 days (14–42 days) for hospital-derived and 27 days (15–42 days) for community-derived MRSA (*p* = 0.8856). The number of deaths during the definitive therapy period was six patients (11%) for the hospital-derived type and four patients (5%) for the community-derived MRSA type (*p* = 0.1977). Table 2B shows the antimicrobial susceptibility results for hospital-derived and community-derived MRSA.

**Table 2A.** Patient characteristics.

		Hospital-derived (n = 54)		Community-derived (n = 81)		*p*-value
Age	Mean (± SD)	65.8	(± 12.8)	70.7	(± 15.0)	0.0478
Sex (male)	N (%)	44	(81.5%)	53	(65.4%)	0.0422
Ambulance used	N (%)	12	(22.2%)	28	(34.6%)	0.1238
Length of hospital stay before specimen submission	Mean (± SD)	32.4	(± 37.3)	25.2	(± 32.8)	0.2515
Barthel Index	Mean (± SD)	68.7	(± 44.7)	56.5	(± 47.1)	0.1304
Charlson Comorbidity Index	Mean (± SD)	2.3	(± 3.55)	2	(± 3.51)	0.6274
Medical practices before submitted specimen						
Antimicrobial administration before submitted specimen	N (%)	51	(94.4%)	73	(90.1%)	0.5249
Duration of antimicrobial therapy before specimen submission	Mean (± SD)	18.6	(± 16.5)	21.8	(± 21.9)	0.3640
Surgery	N (%)	34	(63.0%)	36	(44.4%)	0.0349
Chemotherapy	N (%)	13	(24.0%)	9	(11.1%)	0.0457
Immunosuppressive drugs	N (%)	25	(46.3%)	22	(27.1%)	0.0222
G-CSF preparation	N (%)	6	(11.1%)	2	(2.4%)	0.0592
Blood transfusion	N (%)	15	(27.7%)	27	(33.3%)	0.4946
Central venous catheter use	N (%)	21	(38.8%)	30	(37.0%)	0.8279
Drain use	N (%)	28	(51.8%)	30	(37.0%)	0.0885
Radiotherapy	N (%)	6	(11.1%)	2	(2.4%)	0.0592
Dialysis	N (%)	8	(14.8%)	5	(6.1%)	0.0954
Bronchoscopy	N (%)	5	(9.3%)	5	(6.1%)	0.5204
ICU admission	N (%)	13	(24.1%)	19	(23.5%)	0.9342
Reasons for hospitalization						
Intestinal infectious disease	N (%)	0	(0%)	5	(6%)	
Malignant neoplasms	N (%)	24	(44%)	18	(22%)	
Endocrine, nutritional or metabolic disease	N (%)	4	(7%)	6	(7%)	
Mental or behavioral disorders	N (%)	1	(2%)	1	(1%)	
Disease of the nervous system	N (%)	0	(0%)	5	(6%)	
Disease of the circulatory system	N (%)	9	(17%)	24	(30%)	
Disease of the digestive system	N (%)	6	(11%)	4	(5%)	
Disease of the skin or subcutaneous tissue	N (%)	2	(4%)	6	(7%)	
Disease of the musculoskeletal system or connective tissue	N (%)	5	(9%)	5	(6%)	
Disease of the genitourinary system	N (%)	1	(2%)	4	(5%)	
Injury, poisoning or certain other consequences of external causes	N (%)	2	(4%)	3	(4%)	
MRSA-detected specimens						
Blood	N (%)	2	(3.8%)	6	(7.5%)	
Respiratory	N (%)	35	(64.9%)	41	(50.7%)	
Skin	N (%)	13	(24.1%)	18	(22.3%)	
Urine	N (%)	2	(3.8%)	15	(18.6%)	
Stool	N (%)	1	(1.9%)	0	(0%)	
Central venous catheter	N (%)	1	(1.9%)	1	(1.3%)	

*Note: Abbreviations: G-CSF, Granulocyte-colony stimulating factor; ICU, Intensive care unit; MRSA, Methicillin-resistant *Staphylococcus aureus*.

**Table 2B.** Results of antimicrobial susceptibility of MRSA for the hospital-derived and community-derived groups.

	Hospital-derived				Community-derived			
	Number of patients (N)	S (%)	I (%)	R (%)	Number of patients (N)	S (%)	I (%)	R (%)
VCM	54	100	0	0	81	100	0	0
TEIC	54	100	0	0	81	100	0	0
LZD	54	100	0	0	81	100	0	0
DAP	45	98	0	2	61	100	0	0
CLDM	54	0	0	100	81	30	1	69
LVFX	54	0	0	100	81	10	0	90
MINO	54	15	9	76	81	93	4	4

*Note: Since susceptibility testing for DAP began in 2017, there were no records for patients prior to that time. Abbreviations: VCM, vancomycin; TEIC, teicoplanin; LZD, linezolid; DAP, daptomycin; CLDM, clindamycin; LVFX, levofloxacin; MINO, minocycline. S, susceptible; I, Intermediate; R, Resistant.

### 3.2. Comparison of medical practices

MRSA-related medical practices were identified as follows: examination (e.g., microbiological examination and hematological examination), imaging (e.g., X-ray and computed tomography diagnosis), injection, medical management (e.g., therapeutic drug monitoring), medication and procedure (e.g., sputum suction and artificial respiration).

**Figure 2.**
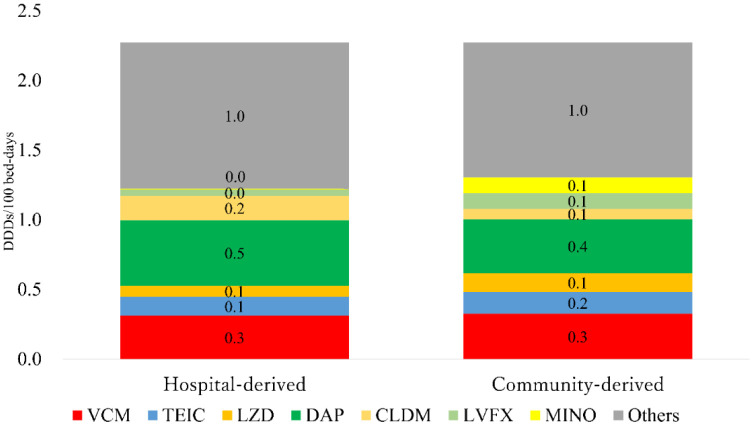
Descriptive AUD for the definitive therapy period. Abbreviations: AUD, antimicrobial use density; VCM, vancomycin; TEIC, teicoplanin; LZD, linezolid; DAP, daptomycin; CLDM, clindamycin; LVFX, levofloxacin; MINO, minocycline.

As seen in Table 3, there was no significant difference in the average number of medical practices during the definitive therapy period among the two groups. The AUDs for the empirical therapy period were 14.1 and 16.1 (per 100 bed-days) for hospital-derived and community-derived MRSA types, respectively. Figure 2 shows the differences in AUD between the two groups for each antibiotic type during the definitive therapy period. The AUDs for the definitive therapy period were 2.3 and 2.3 (per 100 bed-days), respectively, and those for anti-MRSA drugs were 1.0 and 1.0 (per 100 bed-days), respectively. Particularly, hospital-derived clindamycin (CLDM) showed a higher AUD (greater by 0.1 per 100 bed-days) than community-derived CLDM. On the other hand, community-derived levofloxacin (LVFX) and minocycline (MINO) showed a higher AUD (greater by 0.1 per 100 bed-days) than their hospital-derived counterparts.

**Table 3.** Average number of medical practices performed during the definitive therapy period (times / patient).

	Hospital-derived	Community-derived	*p*-value
Examination	2.2	1.9	0.4387^b)^
Imaging	1.1	1.1	0.7954^b)^
Injection	0.6	0.7	0.6507^b)^
Medical management	0.0	0.0	0.6197^b)^
Medication	1.2	1.1	0.5499^a)^
Procedure	1.0	0.9	0.5381^b)^

*Note: a) Student's t-test, b) Welch's t-test.

AUD (Defined Daily Doses [DDDs]/100 bed-days)=[Amount of antimicrobial consumption(g)/DDD(g)]/bed-days×100 (1)

### 3.3. Comparison of medical costs

Table 4 shows that the medical costs for the total therapy period were 274,481 and 311,510 JPY/patient for the hospital-derived and community-derived MRSA groups, respectively (*p* = 0.4784). For the definitive therapy period, the hospital-derived and community-derived costs were 237,698 and 269,970 JPY/patient, respectively (*p* = 0.5126). There were no significant differences in the costs related to MRSA-related medical care and antimicrobials, including anti-MRSA drugs, in either therapy period.

**Figure 3.**
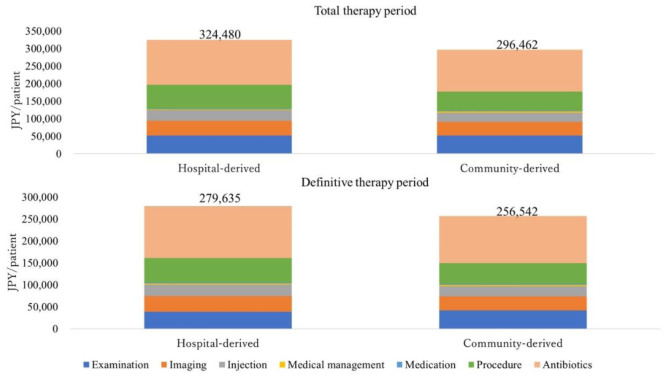
Distribution of costs during the total and definitive therapy periods following IPTW-based analysis.

Figure 3 shows the medical costs after IPTW implementation for the total therapy period (324,480 and 296.462 JPY/patient for the hospital-derived and community-derived MRSA types, respectively; *p* = 0.5813). Significant differences were observed in bacteriological tests (17,923 vs. 10,075 JPY/patient; *p* = 0.0094), nutritional procedures (6,646 vs. 3,901 JPY/patient; *p* = 0.0388), ultrasonography (2,027 vs. 4,835 JPY/patient; *p* = 0.0119) and X-rays (24,909 vs. 18,380 JPY/patient; *p* = 0.0276) during the total therapy period for the hospital-derived and community-derived MRSA infections, respectively (Supplementary Table 2A). For the definitive therapy period, the hospital-derived and the community-derived MRSA types conferred costs of 279,635 and 256,542 JPY/patient, respectively (*p* = 0.6355). Significant differences were observed in the costs of bacteriological examination (12,492 vs. 7,244 JPY/patient; *p* = 0.0470), nutritional procedures (6,224 vs. 3,504 JPY/patient; *p* = 0.0403) and ultrasonography (1,789 vs. 4,041 JPY/patient; *p* = 0.0195) for the hospital-derived and community-derived MRSA types, respectively, during the definitive therapy period (Supplementary Table 2B). No significant differences in medical costs were found for antimicrobials, including anti-MRSA drugs (Table 4). No statistically significant differences in medical costs were observed in MRSA-detected specimens (Supplementary Tables 3A–E).

**Table 4.** Healthcare resource utilization and costs for the unweighted and IPTW-adjusted study population during the total therapy period and definitive therapy period.

	Unweighted (JPY/patient)				IPTW-Adjusted (JPY/patient)			
	Hospital-derived (n = 54)	Community-derived (n = 81)	Mean difference	p-value	Hospital-derived (n = 54)	Community-derived (n = 81)	Mean difference	p-value
Total therapy period								
MRSA-related medical practices and antibiotics	274,481	311,510	-37,029	0.4784	324,480	296,462	28,018	0.5813
MRSA-related medical practices	166,262	182,320	-16,058	0.6496	196,820	177,099	19,721	0.5510
Antibiotics	108,219	129,190	-20,971	0.4674	127,659	119,363	8,296	0.7763
Vancomycin	8,614	9,604	-990	0.7506	8,471	10,702	-2,232	0.4804
Teicoplanin	4,919	6,244	-1,326	0.7259	3,355	5,544	-2,189	0.4893
Linezolid	16,293	31,242	-14,949	0.3813^a)^	12,472	29,902	-17,430	0.2532^a)^
Daptomycin	37,110	34,461	2,649	0.8821	48,331	28,672	19,659	0.3153
								
Definitive therapy period								
MRSA-related medical practices and antibiotics	237,698	269,970	-32,272	0.5126	279,635	256,542	23,093	0.6355
MRSA-related medical practices	137,101	154,433	-17,332	0.5853	160,697	149,895	10,802	0.7170
Antibiotics	100,596	115,537	-14,941	0.5952	118,938	106,647	12,291	0.6689
Vancomycin	8,321	8,719	-398	0.8940	8,301	9,938	-1,637	0.5910
Teicoplanin	4,453	5,380	-928	0.7937	2,938	4,620	-1,682	0.5605
Linezolid	16,293	30,375	-14,083	0.4048^a)^	12,472	29,346	-16,874	0.2657^a)^
Daptomycin	36,860	32,624	4,236	0.8071	48,207	27,158	21,049	0.2731

*Note: Injectable antimicrobial agents classified as “J01” in the Anatomical Therapeutic Chemical Classification Level 3 classification proposed by the World Health Organization as antibiotics in this table. Abbreviations: JPY, Japanese yen; IPTW, Inverse probability of treatment weighting; MRSA, Methicillin-resistant *Staphylococcus aureus*.

## 4. Discussion

In this study, MRSA was classified as hospital- and community-derived types based on POT scores, and medical costs were compared between the two groups. No statistically significant differences were observed in the total medical costs during the total and definitive therapy periods, indicating no significant difference in the cost of infectious disease treatment.

A previous study reported that community-derived MRSA is often observed in relatively young patients [30]. However, patients with hospital-derived MRSA infections in our study tended to be young and were more likely to be immunocompromised, suggesting changes in the distribution of MRSA genes. In recent years, an increase in community-derived MRSA has been reported in nosocomial infections compared to hospital-derived MRSA, and genotypic changes in MRSA strains that cause nosocomial infections have been predicted to occur nationwide [31]. A similar report from the Yamanashi Prefecture suggested the occurrence of a change in genotype distribution among patients with nosocomial infections [32].

There were no significant differences in the frequency of MRSA-related medical practices during the definitive therapy period. Furthermore, this study showed that the use of antimicrobial agents was similar in both groups. The results showed a higher AUD for CLDM in the hospital-derived group and higher AUDs for LVFX and MINO in the community-derived group. These findings were observed from the drug selection we performed based on MRSA susceptibility testing. Following confirmation of the MRSA infection, the same treatment was administered regardless of the genotype, suggesting that the genotype has little effect on the treatment. Accordingly, the hospital length of stay and mortality did not differ between the groups.

Overall, medical costs did not differ significantly between the two groups throughout the total or definitive therapy periods. Medical costs tended to be higher for the hospital-derived MRSA type than the community-derived MRSA type after adjusting for patient characteristics, and it is possible that the medical costs reflected treatment-related factors, such as bacteriological tests and nutritional procedures, for more severe conditions. However, some reports indicate that community-derived MRSA remains susceptible to a relatively high number of antimicrobial agents [33]. The cost burden of antimicrobial agents is expected to increase because MRSA infections are treated over a prolonged period. In this study, antimicrobial agents conferred approximately 40% of the medical costs during the definitive therapy period. The selection of narrow and low-cost antimicrobial agents based on susceptibility results and patient factors may reduce the MRSA-associated economic burden.

This study had several limitations. First, as the analysis was based on a single-center cohort, the small sample may have limited the power to detect intergroup differences. Furthermore, the outcomes of MRSA infection improve with the intervention of infectious disease professionals [34]. Therefore, the results are likely to be strongly influenced by treatment patterns at each facility. In the future, to enhance generalizability, multicenter studies that combine DPC and bacteriology data (including the POT score) and analysis performed at the patient level are needed to obtain conclusive results. Second, the DPC data do not include laboratory findings and vital signs, such as fever or inflammatory response. Thus, a patient with positive bacterial results does not necessarily imply that one has an infectious disease. Therefore, we defined patients as those who had been treated with antimicrobials for a certain period of time. Third, the University of Yamanashi Hospital did not perform PVL testing for MRSA. As such, the presence or absence of PVL-producing bacteria could not be included in the analysis. Lastly, the use of IPTW increased the precision of estimated treatment effects by creating a pseudo population that limited the association between confounders and treatment. Stabilized weights increased precision in the estimates by reducing the variance of the weights. However, due to the observational nature of the study, there was always a possibility that there might be unmeasured confounding factors, thereby leading to residual bias.

## 5. Conclusions

We classified MRSA according to the POT scores and examined their impact on healthcare costs. There were no significant differences in medical costs between hospital-derived and community-derived MRSA infections. In the future, multicenter studies should be conducted to ascertain the economic burden associated with hospital-derived and community-derived MRSA infections.
