# Supplementary material for: Estimated medical costs of methicillin-resistant Staphylococcus aureus infection classified by polymerase chain reaction-based open reading frame typing in Japan
Source: AIMS Microbiol. 2022 Dec 19;8(4):528–43. doi: 10.3934/microbiol.2022034 (PMC9834076; doi:10.3934/microbiol.2022034)
Supplement: Supplementary file 1 [file microbiol-08-04-034-s001.pdf]

AIMS Microbiology

AIMS Microbiology, 8(4): 528–543.
DOI: 10.3934/microbiol.2022034
Received: 14 September 2022
Revised: 25 November 2022
Accepted: 08 December 2022
Published: 19 December 2022

http://www.aimspress.com/journal/microbiology

Research article

Estimated medical costs of methicillin-resistant *Staphylococcus aureus* infection classified by polymerase chain reaction-based open reading frame typing in Japan

Tomokazu Shoji[1,2,3,*], Ryusei Muto[2], Ryoko Sakai[2], Hiroki Matsumura[1,3], Takashi Uchida[3,4], Fumihiko Kitta[1], Osamu Inoue[3], Keishi Kawata[1] and Manabu Akazawa[2]

[1] Department of Pharmacy, University of Yamanashi Hospital, 1110, Shimokato, Chuo, Yamanashi 409–3898, Japan
[2] Department of Public Health and Epidemiology, Meiji Pharmaceutical University, 2–522–1, Noshio, Kiyose, Tokyo 204–8588, Japan
[3] Division of Infection Control and Prevention, University of Yamanashi Hospital, 1110, Shimokato, Chuo, Yamanashi 409–3898, Japan
[4] Department of Clinical Laboratory, University of Yamanashi Hospital, 1110, Shimokato, Chuo, Yamanashi 409–3898, Japan

* **Correspondence:** Email: tshohji@yamanashi.ac.jp; Tel: +81552731111; Fax: +81552736672.

Supplementary

Table S1. POT profiles of 138 isolated MRSA.

POT index	Categorized	Number of patients
93-137-103	Hospital-derived	7
93-137-39	Hospital-derived	1
93-153-111	Hospital-derived	2
93-153-16	Hospital-derived	3
93-155-47	Hospital-derived	1
93-159-117	Hospital-derived	1
93-163-40	Hospital-derived	1
93-171-56	Hospital-derived	1
93-182-109	Hospital-derived	1
93-183-61	Hospital-derived	1
93-190-110	Hospital-derived	1
93-190-34	Hospital-derived	2
93-190-59	Hospital-derived	1
93-191-103	Hospital-derived	20
93-191-39	Hospital-derived	4
93-191-47	Hospital-derived	1
93-217-111	Hospital-derived	5
93-254-59	Hospital-derived	1
106-109-121	Community-derived	1
106-111-113	Community-derived	1
106-129-1	Community-derived	1
106-129-5	Community-derived	5
106-137-35	Community-derived	1
106-137-80	Community-derived	7
106-145-120	Community-derived	1
106-147-101	Community-derived	2
106-149-57	Community-derived	1
106-167-101	Community-derived	1
106-167-16	Community-derived	1
106-167-96	Community-derived	1
106-177-37	Community-derived	1
106-179-13	Community-derived	1
106-179-37	Community-derived	1
106-183-32	Community-derived	4
106-183-33	Community-derived	15
106-183-37	Community-derived	10
106-183-57	Community-derived	1
106-221-113	Community-derived	1
106-247-33	Community-derived	1
106-247-57	Community-derived	1

Continued on next page

POT index	Categorized	Number of patients
106-55-37	Community-derived	1
106-59-112	Community-derived	1
106-59-120	Community-derived	1
106-61-112	Community-derived	1
106-61-34	Community-derived	1
106-77-113	Community-derived	14
106-9-80	Community-derived	2
108-64-97	Community-derived	1

Table S2A. Healthcare resource utilization and costs for the unweighted and IPTW-adjusted study population during the total therapy period.

	Unweighted (JPY/patient)			IPTW Adjusted (JPY/patient)		
	Hospital-derived (n = 54)	Community-derived (n = 81)	*p*-value	Hospital-derived (n = 54)	Community-derived (n = 81)	*p*-value
TDM	2,374	3,045	0.4116 a)	2,368	3,231	0.2678
Microbiological examination	15,326	11,047	0.1592 a)	17,923	10,075	0.0094
Hematological examination	9,620	13,219	0.2321	12,785	13,396	0.848
Electro-encephalography	524	548	0.9629 a)	1,394	472	0.1086
Computed tomography diagnosis	14,967	19,580	0.1465	16,083	20,244	0.2175 a)
First Aid	28,750	31,389	0.8641 a)	38,250	29,100	0.551
Ophthalmology examination	132	349	0.2921	92	493	0.0894
Dermatology procedures	1,731	456	0.0902	1,240	454	0.2482
Injection charges	23,021	26,702	0.4739 a)	29,853	25,344	0.3691 a)
Immunological tests	7,821	11,044	0.154	8,984	12,450	0.1449
Vital monitoring	3,067	5,014	0.1031 a)	4,445	4,594	0.9146
Nuclear medicine diagnosis	1,000	667	0.7659	1,234	553	0.5711
Aseptic preparation	807	790	0.9617 a)	682	728	0.8863 a)
Endoscopy	2,398	3,498	0.4605	1,756	3,391	0.251
Nutritional procedures	5,656	4,215	0.325 a)	6,946	3,901	0.0388
Urine and fecal examination	1,883	1,873	0.9846 a)	2,029	2,155	0.8302 a)
Prescription	1,962	2,003	0.8176 a)	2,225	2,040	0.2944
General procedures	22,507	21,636	0.9438 a)	21,230	20,641	0.9538 a)
Psychological tests	83	0	0.3219	63	0	0.3897
Ultrasonography	1,980	5,109	0.0067	2,027	4,835	0.0119
Urological procedures	141	257	0.0321	245	224	0.7226
X-ray	20,371	19,507	0.7706 a)	24,909	18,380	0.0276
Pharmaceutical service	141	375	0.2103	60	399	0.0682 a)

*Note: Comparison of the two groups was tested using Student's t-test. a) Welch's t-test. Abbreviations: TDM, Therapeutic drug monitoring.

Table S2B. Healthcare resource utilization and costs for the unweighted and IPTW-adjusted study population during the definitive therapy period.

	Unweighted (JPY/patient)				IPTW Adjusted (JPY/patient)			
	Hospital-derived (n = 54)	Community-derived (n=81)	p-value		Hospital-derived (n=54)	Community-derived (n=81)	p-value	
TDM	2,374	2,850	0.5369	a)	2,368	3,062	0.3435	a)
Microbiological examination	10,687	7,937	0.2906	a)	12,492	7,244	0.0389	
Hematological examination	7,852	10,627	0.3		10,155	10,466	0.9009	
Electro-encephalography	380	385	0.9896		281	350	0.8579	
Computed tomography diagnosis	11,457	15,015	0.2514	a)	13,971	16,058	0.5301	a)
First Aid	24,563	27,423	0.8351	a)	29,883	25,346	0.7364	a)
Ophthalmology examination	132	321	0.4278		92	472	0.1229	
Dermatology procedures	1,579	365	0.0424		1,130	361	0.1812	
Injection charges	19,510	23,261	0.4475		25,498	22,047	0.4586	a)
Immunological tests	6,421	9,113	0.2535		7,260	10,411	0.1654	
Vital monitoring	2,717	4,188	0.1931	a)	4,220	3,861	0.7828	
Nuclear medicine diagnosis	1,000	444	0.5719		1,234	365	0.4352	
Aseptic preparation	778	726	0.8785	a)	663	666	0.9927	a)
Endoscopy	1,613	3,201	0.2865		1,095	3,037	0.1289	
Nutritional procedures	5,089	3,793	0.3352	a)	6,224	3,504	0.0334	
Urine and fecal examination	1,547	1,625	0.8787	a)	1,569	1,904	0.5427	a)
Prescription	1,734	1,780	0.7933	a)	1,986	1,822	0.3346	
General procedures	18,557	19,936	0.908	a)	18,259	18,590	0.9733	a)
Psychological tests	83	0	0.222		63	0	0.3502	
Ultrasonography	1,556	4,247	0.0138		1,789	4,041	0.0224	
Urological procedures	126	227	0.0425	a)	236	201	0.5236	
X-ray	17,207	16,593	0.8164	a)	20,168	15,688	0.0778	a)
Pharmaceutical service	141	375	0.2656		60	399	0.08	

*Note: Comparison of the two groups was tested using Student's t-test. a) Welch's t-test. Abbreviations: TDM, Therapeutic drug monitoring.

Table S3A. Healthcare resource utilization and costs for the unweighted and IPTW-adjusted study population by blood specimens.

	Unweighted (JPY/patient)				IPTW Adjusted (JPY/patient)			
	Hospital-derived (n = 2)	Community-derived (n = 6)	Mean difference	p-value	Hospital-derived (n = 2)	Community-derived (n = 6)	Mean difference	p-value
Total therapy period								
MRSA-related medical practices and antibiotics	442,360	283,305	159,055	0.6025	432,497	337,854	94,643	0.7727
MRSA-related medical practices	176,225	85,737	90,488	0.5718	173,136	89,905	83,231	0.5975
Antibiotics	281,145	225,109	56,036	0.8255	274,670	274,377	293	0.9992
Vancomycin	16,261	14,551	1,709	0.9001	16,452	12,804	3,648	0.7835
Teicoplanin	62,089	12,978	49,111	0.1966	60,406	12,858	47,548	0.2111
Linezolid	133,399	133,399	0	1	129,783	170,435	-40,652	0.8463
Daptomycin	0	33,825	-33,825	0.4212	0	41,497	-41,497	0.3491
Definitive therapy period								
MRSA-related medical practices and antibiotics	435,226	254,399	180,827	0.5573	425,360	312,430	112,930	0.7338
MRSA-related medical practices	161,215	58,197	103,018	0.5602	157,828	63,478	94,350	0.5874
Antibiotics	274,011	196,202	77,809	0.7601	267,532	248,953	18,579	0.9471
Vancomycin	15,775	9,888	5,888	0.6425	15,953	8,874	7,079	0.5617
Teicoplanin	62,089	8,807	53,283	0.145	60,406	8,875	51,531	0.1581
Linezolid	133,399	121,697	11,702	0.9502	129,783	160,608	-30,825	0.8824
Daptomycin	0	33,825	-33,825	0.4212	0	41,497	-41,497	0.3491

Table S3B. Healthcare resource utilization and costs for the unweighted and IPTW-adjusted study population by respiratory specimens.

	Unweighted (JPY/patient)				IPTW Adjusted (JPY/patient)			
	Hospital-derived (n = 35)	Community-derived (n = 41)	Mean difference	p-value	Hospital-derived (n = 35)	Community-derived (n = 41)	Mean difference	p-value
Total therapy period								
MRSA-related medical practices and antibiotics	233,240	330,691	-97,451	0.185	265,582	308,592	-43,010	0.5378
MRSA-related medical practices	198,935	251,675	-52,740	0.3581	224,429	237,180	-12,751	0.8072
Antibiotics	68,529	112,345	-43,816	0.2014	84,489	102,695	-18,206	0.5598
Vancomycin	11,495	12,237	-741	0.8807	11,088	11,452	-364	0.9368
Teicoplanin	0	4,231	-4,231	0.0623	0	3,433	-3,433	0.0527
Linezolid	12,084	32,611	-20,527	0.3512	12,060	31,778	-19,718	0.3325
Daptomycin	3,136	8,250	-5,114	0.4166	3,598	6,522	-2,924	0.582
Definitive therapy period								
MRSA-related medical practices and antibiotics	225,677	319,759	-94,082	0.2113	256,283	298,025	-41,742	0.5481
MRSA-related medical practices	164,711	218,346	-53,635	0.2962	181,093	205,898	-24,805	0.6004
Antibiotics	60,966	101,413	-40,447	0.228	75,190	92,127	-16,937	0.5819
Vancomycin	11,071	11,436	-365	0.9382	10,863	10,685	177	0.9677
Teicoplanin	0	3,604	-3,604	0.0769	0	2,906	-2,906	0.0657
Linezolid	12,084	32,611	-20,527	0.3512	12,060	31,778	-19,718	0.3325
Daptomycin	2,749	8,250	-5,501	0.3804	3,424	6,522	-3,098	0.5583

Table S3C. Healthcare resource utilization and costs for the unweighted and IPTW-adjusted study population by skin specimens.

	Unweighted (JPY/patient)				IPTW Adjusted (JPY/patient)			
	Hospital-derived (n = 13)	Community-derived (n = 18)	Mean difference	p-value	Hospital-derived (n = 13)	Community-derived (n = 18)	Mean difference	p-value
Total therapy period								
MRSA-related medical practices and antibiotics	211,825	279,033	-67,208	0.4862	267,187	237,798	29,389	0.7708
MRSA-related medical practices	81,767	102,530	-20,763	0.5615	98,667	97,548	1,119	0.9750
Antibiotics	149,256	190,984	-41,728	0.5895	186,400	153,914	32,486	0.6753
Vancomycin	2,331	8,424	-6,094	0.3139	1,561	15,506	-13,945	0.0674
Teicoplanin	4,241	3,729	512	0.9288	5,199	4,201	998	0.8701
Linezolid	14,621	21,063	-6,442	0.8184	9,666	15,682	-6,015	0.7995
Daptomycin	97,832	125,009	-27,177	0.6610	139,678	93,722	45,956	0.4855
Definitive therapy period								
MRSA-related medical practices and antibiotics	204,352	263,941	-59,589	0.5182	259,300	225,880	33,420	0.7325
MRSA-related medical practices	62,569	88,048	-25,479	0.4583	80,787	83,884	-3,097	0.9293
Antibiotics	141,783	175,893	-34,110	0.6350	178,514	141,996	36,518	0.6192
Vancomycin	2,331	8,297	-5,967	0.3156	1,561	15,216	-13,656	0.0671
Teicoplanin	3,780	3,729	51	0.9926	4,634	4,201	433	0.9407
Linezolid	14,621	21,063	-6,442	0.8184	9,666	15,682	-6,015	0.7995
Daptomycin	97,832	116,741	-18,909	0.7462	139,678	87,548	52,130	0.4114

Table S3D. Healthcare resource utilization and costs for the unweighted and IPTW-adjusted study population by stool specimens.

	Unweighted (JPY/patient)				IPTW Adjusted (JPY/patient)			
	Hospital-derived (n = 1)	Community-derived (n = 0)	Mean difference	p-value	Hospital-derived (n = 1)	Community-derived (n = 0)	Mean difference	p-value
Total therapy period								
MRSA-related medical practices and antibiotics	248,736	-	248,736	N/A	-	-	-	N/A
MRSA-related medical practices	140,520	-	140,520	N/A	-	-	-	N/A
Antibiotics	108,216	-	108,216	N/A	-	-	-	N/A
Vancomycin	0	-	0	N/A	-	-	-	N/A
Teicoplanin	86,262	-	86,262	N/A	-	-	-	N/A
Linezolid	0	-	0	N/A	-	-	-	N/A
Daptomycin	0	-	0	N/A	-	-	-	N/A
Definitive therapy period								
MRSA-related medical practices and antibiotics	195,258	-	195,258	N/A	-	-	-	N/A
MRSA-related medical practices	114,570	-	114,570	N/A	-	-	-	N/A
Antibiotics	80,688	-	80,688	N/A	-	-	-	N/A
Vancomycin	0	-	0	N/A	-	-	-	N/A
Teicoplanin	67,116	-	67,116	N/A	-	-	-	N/A
Linezolid	0	-	0	N/A	-	-	-	N/A
Daptomycin	0	-	0	N/A	-	-	-	N/A

*Note: N/A, not applicable.

Table S3E. Healthcare resource utilization and costs for the unweighted and IPTW-adjusted study population by central venous catheter specimens.

	Unweighted (JPY/patient)				IPTW Adjusted (JPY/patient)			
	Hospital-derived (n = 1)	Community-derived (n = 1)	Mean difference	p-value	Hospital-derived (n = 1)	Community-derived (n = 1)	Mean difference	p-value
Total therapy period								
MRSA-related medical practices and antibiotics	491,788	105,584	386,204	N/A	506,148	114,744	391,404	N/A
MRSA-related medical practices	163,440	107,520	55,920	N/A	163,440	107,520	55,920	N/A
Antibiotics	342,708	7,224	335,484	N/A	342,708	7,224	335,484	N/A
Vancomycin	0	0	0	N/A	0	0	0	N/A
Teicoplanin	0	0	0	N/A	0	0	0	N/A
Linezolid	0	0	0	N/A	0	0	0	N/A
Daptomycin	270,600	0	270,600	N/A	270,600	0	270,600	N/A
Definitive therapy period								
MRSA-related medical practices and antibiotics	489,596	105,068	384,528	N/A	489,596	105,068	384,528	N/A
MRSA-related medical practices	149,080	98,360	50,720	N/A	149,080	98,360	50,720	N/A
Antibiotics	340,516	6,708	333,808	N/A	340,516	6,708	333,808	N/A
Vancomycin	0	0	0	N/A	0	0	0	N/A
Teicoplanin	0	0	0	N/A	0	0	0	N/A
Linezolid	0	0	0	N/A	0	0	0	N/A
Daptomycin	270,600	0	270,600	N/A	270,600	0	270,600	N/A

*Note: N/A, not applicable.

AIMS Press

© 2022 the Author(s), licensee AIMS Press. This is an open access article distributed under the terms of the Creative Commons Attribution License (http://creativecommons.org/licenses/by/4.0)
